# Lung regeneration: diverse cell types and the therapeutic potential

**DOI:** 10.1002/mco2.494

**Published:** 2024-02-23

**Authors:** Yutian Chen, Zhen Li, Gaili Ji, Shaochi Wang, Chunheng Mo, Bi‐Sen Ding

**Affiliations:** ^1^ The Department of Endovascular Surgery The First Affiliated Hospital of Zhengzhou University Zhengzhou China; ^2^ Key Laboratory of Birth Defects and Related Diseases of Women and Children of MOE, State Key Laboratory of Biotherapy, West China Second University Hospital, Sichuan University Chengdu China; ^3^ Department of Gynecology The Third Affiliated Hospital of Zhengzhou University Zhengzhou China; ^4^ Department of Translational Medicine The First Affiliated Hospital of Zhengzhou University Zhengzhou China

**Keywords:** cellular composition, lung regeneration, molecular mechanisms, research models, therapeutic potential

## Abstract

Lung tissue has a certain regenerative ability and triggers repair procedures after injury. Under controllable conditions, lung tissue can restore normal structure and function. Disruptions in this process can lead to respiratory system failure and even death, causing substantial medical burden. The main types of respiratory diseases are chronic obstructive pulmonary disease (COPD), idiopathic pulmonary fibrosis (IPF), and acute respiratory distress syndrome (ARDS). Multiple cells, such as lung epithelial cells, endothelial cells, fibroblasts, and immune cells, are involved in regulating the repair process after lung injury. Although the mechanism that regulates the process of lung repair has not been fully elucidated, clinical trials targeting different cells and signaling pathways have achieved some therapeutic effects in different respiratory diseases. In this review, we provide an overview of the cell type involved in the process of lung regeneration and repair, research models, and summarize molecular mechanisms involved in the regulation of lung regeneration and fibrosis. Moreover, we discuss the current clinical trials of stem cell therapy and pharmacological strategies for COPD, IPF, and ARDS treatment. This review provides a reference for further research on the molecular and cellular mechanisms of lung regeneration, drug development, and clinical trials.

## Graphical abstract

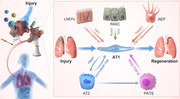

## 1 INTRODUCTION

As the aging population rapidly grows, pulmonary diseases such as idiopathic pulmonary fibrosis (IPF), chronic obstructive pulmonary disease (COPD), and asthma place a substantial burden on the public health system.^1^, ^2^, ^3^, ^4^, ^5^ Currently, chronic respiratory diseases are the third leading cause of death, responsible for 4.0 million deaths, with a prevalence of 454.6 million cases globally.^6^ With socialization and industrialization, the burden of disease has transitioned from acute and subacute infectious diseases to a chronic noncommunicable disease pattern, and even with the recent coronavirus disease 2019 (COVID‐19) pandemic, a significant proportion of patients recovering from acute infectious diseases have moved to chronic noncommunicable clinical syndromes. Many respiratory diseases, especially IPF, are accompanied by varying degrees of fibrotic response, characterized by progressive scarring of lung tissue, which reduces gas exchange and leads to high disability and mortality worldwide.^7^, ^8^

The lungs are in direct contact with the outside world, and adults are susceptible to lung injury as they inhale more than 10,000 L of unsterile air daily.^9^ Many factors, such as environmental pollution, smoking, viral infections, and autoimmune diseases, contribute to lung injury.^10^ The lungs have a certain regenerative capacity and initiate a repair program after injury. Young individuals have a higher probability of recovering normal lung structure and function than adults after acute injury. However, after the age of 35 years, the ability to recover lung function significantly diminishes. Especially after chronic injury, incomplete and inefficient repair of damaged tissues usually leads to varying degrees of fibrosis.^2^, ^11^ There is no effective treatment for organ fibrosis. To date, the United States Food and Drug Administration (US FDA) has only approved two drugs for the treatment of IPF, pirfenidone and nintedanib, but these two drugs can only slow down the progression of the disease and cannot completely cure or reverse pulmonary fibrosis.^12^, ^13^, ^14^ The lungs are a dynamic and multicellular organ, and multiple cell types, such as pulmonary epithelial cells, vascular endothelial cells (ECs), airway epithelial cells, fibroblasts, macrophages, neutrophils, and platelets, work together in the repair process following injury. Different cytokines and signaling pathways, such as WNT, Notch, transforming growth factor (TGF), and platelet derived growth factor (PDGF), are involved in the regulation of lung regeneration and fibrosis. A deeper understanding of the molecular and cellular mechanisms underlying lung regeneration and fibrosis could contribute to the discovery of new effective therapeutic targets and the design of new treatment strategies.^2^, ^15^, ^16^, ^17^

In this review, we summarize the different cell types involved in lung regeneration and fibrosis, as well as the molecular mechanisms and signaling pathways involved in this process. We also present some of the animal models and in vitro 3D culture models for studying lung diseases. For IPF, COPD, and acute respiratory distress syndrome (ARDS), we present some completed and ongoing clinical trials on stem cell transplantation, point out some current issues in the treatment of lung diseases with stem cell transplantation, and propose future development directions.

## 2 ROLE OF SPECIFIC CELL TYPES IN LUNG REGENERATION

**FIGURE 1:**
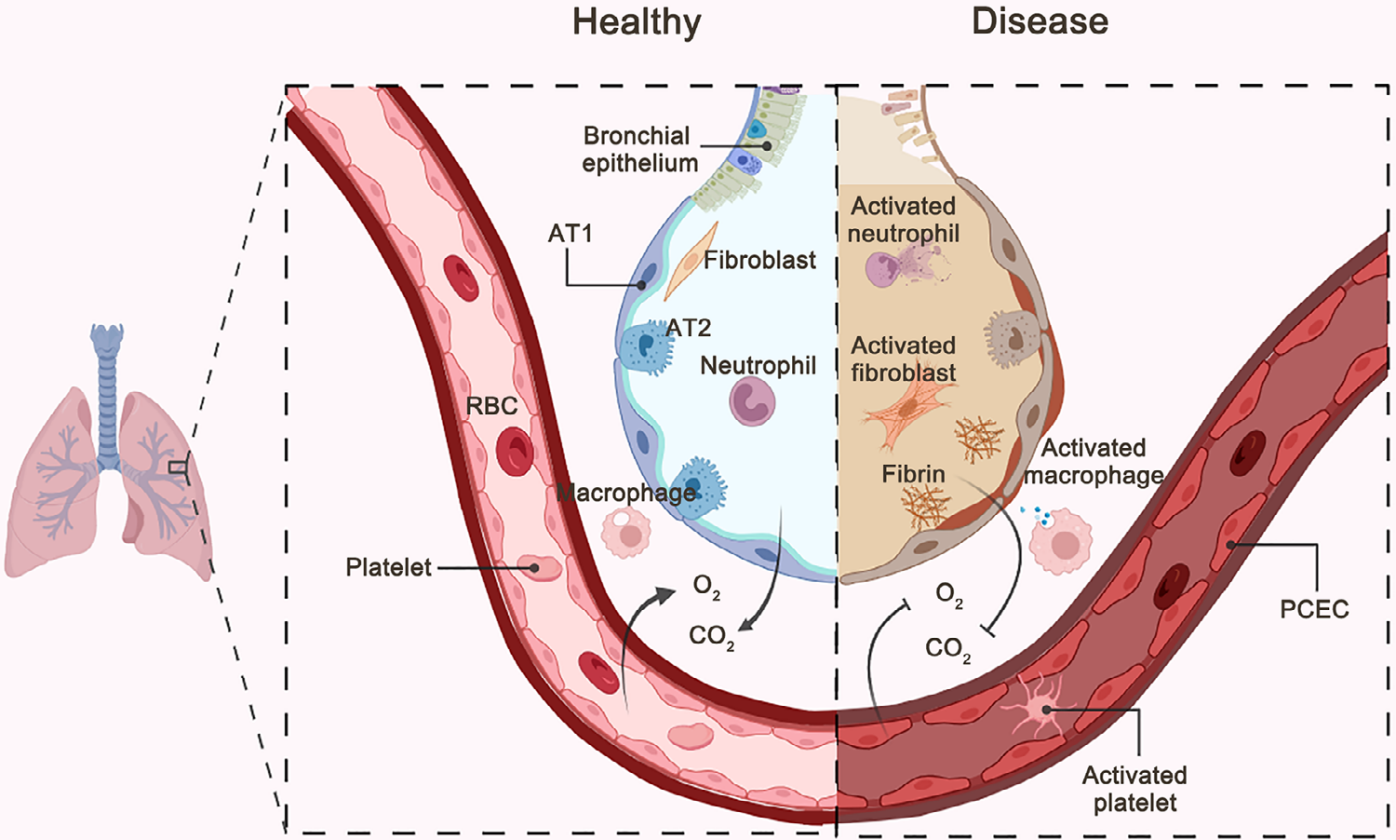
The balance of the lung repair process. Under homeostatic conditions, various cells in the lungs work in an orderly manner and perform gas exchange functions normally. Upon injury, a variety of cells are activated, such as fibroblasts, immune cells, and epithelial cells, triggering an inflammatory response and secretion of extracellular matrix, which ultimately leads to impaired gas exchange. AT1: type I alveolar epithelial cell, AT2: type II alveolar epithelial cell; PCEC, pulmonary capillary endothelial cell; RBC, red blood cell.

The lungs are a multicellular organ. Various cells act synergistically to participate in the repair process following injury. After injury, immune cells are quickly recruited by cytokines secreted by vascular ECs and lung epithelial cells to the damaged site for repair. This process is accompanied by the activation of fibroblasts, their differentiation into myofibroblasts, and secretion of extracellular matrix (ECM). Type 2 lung epithelial cells and airway epithelial cells in the respiratory system, as progenitor/precursor cells, quickly enter the differentiation process to supplement damaged type 1 alveolar epithelial cells and maintain normal lung function (Figure 1). Next, we introduce several cell types involved in the repair of lung injury.

### 2.1 Alveolar epithelial cells

The executive function units of the lungs are alveoli, which are mainly composed of type I alveolar epithelial cells (AT1 cells) and type 2 alveolar epithelial cells (AT2 cells). AT1 cells cover 95% of the alveolar surface area; they are thin and interface with pulmonary capillary ECs (PCECs). AT1 cells, which are marked by aquaporin 5 (AQP5^+^), podoplanin (PDPN^+^), and HOP homeobox (HOPX^+^), facilitate the process of gas exchange.^18^ AT2 cells, which are marked by surfactant protein C (SFTPC^+^), are of cuboidal shape.^19^ They are responsible for generating lung surfactant, which is crucial for reducing the surface tension of the alveolar surface area to protect the lungs from collapsing upon every breath. Lineage‐tracing experiments have shown that AT2 cells act as stem/progenitor cells and can self‐renew and transdifferentiate to AT1 cells. After injury, AT2 cells enter a stepwise repair process, including proliferation, partial de‐differentiation, transition into an AT2–AT1 intermediate state, and finally differentiation. Under normal circumstances, the lung epithelium is in a stationary state and immediately enters the cell proliferation cycle after stimulation, thereby undergoing expansion. Induced by different cytokines (hepatocyte growth factor [HGF] and sphingosine‐1‐phosphate [S1P]), the lung epithelium differentiates into AT1 cells to maintain lung function.^20^

Diverse in vivo injury models of mice and in vitro 3D organoid culture models have revealed the transient AT2 cell subpopulations that appear in the middle of the transition from AT2 to AT1, named as “alveolar differentiation intermediate (ADI),” “pre‐alveolar type‐1 transitional cell state (PATS),” “damage‐associated transitional progenitors (DATPs),” or “AT0” by different groups.^19^, ^21^, ^22^, ^23^ The expression profiles of these cells are somewhat similar; for example, most of them express keratin 8 (Krt8), while some also express claudin 4 (Cldn4), keratin 19 (Krt19), connective tissue growth factor (Ctgf), and stratifin (Sfn).^22^ AT2 cells in regenerating lung models transiently acquire an intermediate state from which they can differentiate into AT1 cells or terminal and respiratory bronchiole secretory cells.^24^ Another study has shown that AT2 cells undergoing differentiation into AT1 cells acquire PATS en route to terminal maturation.^22^ Single‐cell RNA sequencing in combination with in vivo lineage tracing has indicated that interstitial macrophage‐derived interleukin 1 beta (IL‐1β) primes a subset of AT2 cells expressing *interleukin 1 receptor type 1* (*Il1r1*) for conversion into DATPs by *hypoxia inducible factor 1 subunit alpha* (*HIF1α*)‐associated glycolysis pathway, which is required for mature AT1 cell differentiation.^21^ In most cases, AT2 cell proliferation is a regenerative response after injury. However, uncontrolled AT2 cell proliferation can also be pathological and may lead to hyperplasia and, consequently, adenocarcinoma.^25^ Ineffectual AT2‐to‐AT1 differentiation may contribute to the failure of the lung repair regeneration program in patients with IPF. Single‐cell RNA‐sequencing study of lineage‐labeled AT2 cells has indicated two different transitional states of AT2‐to‐AT1 differentiation, early and late. Krt 8 is highly upregulated during early differentiation and downregulated during the late stage. Krt 8^+^ state appears in several independent mouse lung injury models, persists in human lung fibrosis, and may be regulated by the TGF‐β signaling pathway.^19^, ^26^, ^27^ The current research focuses on how to trigger the physiological process of AT2 cell proliferation and transdifferentiation to induce the regeneration and repair of the impaired lung.^22^, ^28^, ^29^, ^30^, ^31^

With the progress of biotechnology, an increasing number of sources of lung tissue progenitor/stem cells have been detected. Respiratory airway secretory (RAS) cells located in human distal airways are unidirectional progenitors for AT2 cells, which are regulated by Notch and Wnt signaling.^32^ Promoting the functional proliferation of alveolar epithelial cells in damaged lung tissue is one of the strategies for developing clinical treatments for lung diseases.

### 2.2 Vascular ECs

There is increasing awareness that blood vessels are not only passive conduits for delivering blood, oxygen, and nutrients, but they also play positive roles in regulating physiological activities.^33^, ^34^, ^35^, ^36^, ^37^, ^38^ ECs attached to the inside of the blood vessel lumen instruct vascular niche by secreting angiocrine factors to sustain peripheral tissue homeostasis. The modalities of angiocrine factors vary and include soluble or membrane‐bound stimulatory and inhibitory factors, exosomes, cytokines, chemokines, and other cellular outcomes produced by tissue‐specific ECs. PCECs deploy diverse angiocrine factors to balance the repair process after lung injury. PCECs are identified by the following expression pattern: platelet and endothelial cell adhesin molecule1 (CD31^+^), CD34^+^, vascular endothelial growth factor receptor 1 (VEGFR1^+^) fibroblast growth factor receptor 1 (FGFR1^+^), and CD45^−^.^39^, ^40^, ^41^ Targeted editing of vascular ECs can modify the vascular niche, make it suitable for the proliferation and differentiation of endogenous or exogenous stem cells, and promote the regeneration of damaged lung tissue. With the recognition of the active regulation by blood vessels, there has been increasing research in recent years on the regulation of pulmonary regeneration by vascular ECs.

Alveolar capillary dysplasia with misalignment of pulmonary veins (ACDMPV), which causes respiratory distress and death in the first month of life, is known as a severe congenital disease that is characterized by diminished lung angiogenesis, which causes scarcity of the alveolar capillary network.^42^, ^43^, ^44^ The transcription factor forkhead box F1 in haploid knockout mouse lung endothelial progenitor cells (EPCs) downregulates the bone morphogenetic protein 9 (BMP9)/activin A receptor like type 1 signaling pathway, inhibits angiogenesis, and aggravates ACDMPV. Treatment with BMP9 restores lung alveolarization and angiogenesis in mice.^45^ Tracheal injection of *Pseudomonas aeruginosa* induces lung injury, resulting in severe alveolar damage and a substantial reduction in the number of AT1 cells. It has been found that S1P secreted by lung microvascular ECs acts on AT2 cells through its receptor, inducing nuclear translocation of yes‐associated protein (YAP) and promoting AT2‐to‐AT1 differentiation, thereby repairing damaged alveoli.^46^ The differentiation process from AT2 to AT1 cells is precisely regulated, with any defect in this process leading to failure in repair and regeneration. AT2 cells are activated by the Notch signaling pathway and differentiated into AT1 cells; however, continued expression of the Notch signaling pathway results in a stagnant differentiation process. This results in the production of a large population of transition‐state cells that express low levels of both AT1 and AT2 markers and fail to fully differentiate into a population of AT1 cells that perform the function of gas exchange. The well‐timed switch‐off of the Notch signaling pathway is mediated by the noncanonical Notch ligand delta‐like 1 homolog, which assists in the completion of the differentiation process from AT2 to AT1 cells.^47^ Volpe et al.^48^ have shown that fms related receptor tyrosine kinase 1 (Flt1), produced by lung ECs, impedes the transdifferentiation of AT2 cells into AT1 cells, having a negative effect on lung repair in pulmonary fibrosis. Another cell type of the vascular system is the vascular smooth muscle cell. Pulmonary arterial hypertension and pulmonary fibrosis are common serious complications of systemic sclerosis, with high overlap. Vascular remodeling caused by excessive growth and migration of vascular smooth muscle cells is an important feature.^49^ Ubiquitin‐specific protease 15 promotes the proliferation and migration of vascular smooth muscle cells in a YAP/TAZ‐dependent manner, thereby regulating the disease progression of pulmonary hypertension.^50^

Single‐cell analysis has elucidated that alveolar endothelium consists of two intermingled cell types, which are similar to the lung epithelium. The first cell type, alveolar capillary cells or aerocytes, is specialized for gas exchange and leukocyte transportation and is specific to the lungs. The other cell type, general capillary, specializes in regulating vasomotor tone and functions as a stem/progenitor cell in capillary homeostasis and repair.^51^ EPCs play an effective role in the treatment of lung diseases. In pneumonia, sepsis‐induced lung injury, and acute lung injury (ALI) models, EPCs can reduce inflammation, mitigate vascular leakage, and potentiate bacterial clearance.^52^, ^53^, ^54^ Intratracheal administration of EPC exosomes reduces lipopolysaccharide (LPS)‐induced ALI at 24 and 48 h.^55^

The lungs are a highly vascularized organ, and dysplastic vasculature leads to severe lung disease, such as ACDMPV. Vascular ECs are structurally tightly integrated with lung epithelial cells and mediate the gas exchange function of the lungs. In addition, vascular ECs and EPCs actively regulate the repair and regeneration process of damaged lung tissues through paracrine and autocrine routes.^56^ Therapeutic modalities for the treatment of lung diseases with vascular ECs are currently under development.

### 2.3 Airway epithelium

Structurally, the proximal part of the human lung consists of the trachea and bronchi, and the distal axis of the bronchi is divided into small bronchioles.^57^ The ciliated pseudostratified epithelium attached to the inside of the airway forms a barrier that acts as a gatekeeper. The integrity of the epithelial barrier of the respiratory system is critical to mammals. After lung injury, the proliferation and migration of airway‐derived stem/progenitor cells are required for lung regeneration and repair.^11^, ^58^ Hypoxia triggers the direct differentiation of airway basal stem cells into solitary neuroendocrine cells, which are epithelial cells with many characteristics of neurons, including the existence of secretory vesicles and the ability to perceive environmental stimuli.^59^

In a fibrotic lung, cytokeratin 5 (KRT5)‐positive basal cells—which are usually present in the conducting airways—extend to the alveolar spaces and line honeycomb cysts.^60^ The presence of KRT5^+^ basal cells in the alveolar region in IPF is associated with increased mortality.^61^ KRT5^+^ cell migration features and expression of remodeling genes are regulated by ECM composition. Overexpression of secreted protein acidic and cysteine rich and ECM glycoprotein in the IPF primary human lung fibroblast matrix limits KRT5^+^ cell migration. Differences in the lung ECM of IPF patients lead to changes in the behavior and function of KRT5^+^ cells, ultimately contributing to remodeling of the lung niche.^62^ Another organoid culture and xenotransplantation study showed that human AT2 cells can be transformed into KRT5^+^ basal cells. This result indicates that human AT2 cells are one of the sources of metaplastic KRT5^+^ basal cells in severe lung injuries such as IPF and COVID‐19‐related pneumonia.^63^ In contrast, KRT5^+^ basal cells in the alveolar region of mice are derived from KRT5^−^/sex‐determining region Y‐box 2 (SOX2)‐positive progenitor cells in mouse airways.^64^, ^65^, ^66^, ^67^

Submucosal glands (SMGs) are located in human or other large mammals’ cartilaginous airways. SMGs remove lung microorganisms and particulate matter by secreting mucus and participate in the regulation of the immune process by producing antimicrobial proteins or peptides. Abnormal mucus secreted from SMGs can contribute to cystic fibrosis (CF). Analysis of SMGs from newborn pigs has shown that the cell types and transcription levels are the same in CF and non‐CF, suggesting that loss of epithelial anion secretion rather than an intrinsic cell defect results in CF mucus abnormalities.^68^

There is a rare cell type in the conducting airways of both humans and mice, namely pulmonary ionocytes. These cells express forkhead box I 1 (FoxI 1) and the CF gene, CFTR. Knockout of FoxI 1 in murine ionocytes results in a loss of Cftr expression and disrupts airway fluid and mucus physiology.^69^, ^70^

### 2.4 Fibroblasts

Fibroblasts, distributed throughout the pulmonary interstitium, play a key role in maintaining lung architecture. They are located between epithelial cells and ECs and usually function normally at a steady state. ECM is in a dynamic balance of synthesis and degradation under normal conditions.^71^ After acute or chronic lung injury, fibroblasts are activated to participate in the repair process by producing ECM. After the repair of acute injury, ECM can be degraded in time, and the normal morphology of the tissue is subsequently restored. However, persistent or overwhelming damage causes excessive deposition of ECM and destroys organ remodeling and normal function.^72^, ^73^ ECs modulate the activation of fibroblasts by producing paracrine factors, including stromal‐cell‐derived factor 1 (SDF1), Jag1, insulin‐like growth factor binding protein 7, and ADAM metallopeptidase with thrombospondin type 1 motif 1. Fibroblasts are recognized as the central mediator of ECM production in lung fibrosis. The activation of fibroblasts is not caused by a single factor, so it is necessary to identify the underlying mechanisms. Fibroblasts exhibit different phenotypes during the formation and resolution of fibrosis. Genetic engineering in mice has indicated the existence of a lipogenic‐to‐myogenic switch in fibroblastic phenotype during the formation of fibrosis; in contrast, there is a myogenic‐to‐lipogenic switch during fibrosis regression.^74^ In addition to secreting ECM, fibroblasts participate in regulating alveolar epithelial cell function by secreting a set of signaling factors to form specific niches. Fibroblasts maintain the proliferative capacity and stem cell identity by providing a short‐range paracrine Wnt signal.^75^ Single‐cell sequencing of human and mouse fibrotic lungs has revealed spatial and temporal heterogeneity of collagen‐producing fibroblasts. Fibroblasts expressing collagen triple‐helix repeat containing 1 (Cthrc1), the marker of pathological fibroblasts in pulmonary fibrosis, express the highest levels of collagen and acquire more migration capabilities than other subsets of collagen‐producing cells. These results suggest that Cthrc1^+^ fibroblasts play a pivotal role in the development of pulmonary fibrosis in humans and mice. The specific source and regulatory mechanism of Cthrc1^+^ fibroblasts still require more direct experimental results, such as in vivo lineage tracing and genetically modified mice.^76^

Myofibroblasts are a further differentiated subset of fibroblasts, which are crucial effector cells in a variety of fibrotic diseases, including IPF. In addition to producing ECM, myofibroblasts acquire the cytoskeletal features of contractile smooth muscle cells by producing α‐smooth muscle actin, which distinguishes them from their precursor fibroblasts.^77^, ^78^ With enhanced contractility, myofibroblasts trigger progressive tissue stiffness, causing a persistent profibrotic stimulus.^79^ In bleomycin‐induced mouse models, BMP4 deficiency perpetuates activation of pulmonary myofibroblasts and leads to accelerated decline in lung function, severe fibrosis, and death. This phenotypic alteration is mediated by TGF. BMP4 reduces fibrosis by decreasing impaired mitochondrial autophagy and cellular senescence in lung fibroblasts and attenuates TGF‐β1‐induced fibroblast differentiation to myofibroblasts and ECM production.^80^ Pericytes are another source of myofibroblasts, and human lung pericytes are activated to transdifferentiate into myofibroblast‐like cells under the regulation of TGF‐β signaling.^81^ In lung fibrosis‐associated diseases, myofibroblasts are derived from a wide range of sources, mainly from the activation of fibroblasts and also from epithelial‐to‐mesenchymal transition (EMT) and/or recruitment and maturation of circulating pluripotent stem mesenchymal progenitor cells known as fibrocytes.^82^, ^83^

### 2.5 Macrophages

Pulmonary fibrosis is usually accompanied by immune dysregulation. The lung microenvironment contains a variety of immune cells, which participate in the repair process. Macrophages, the most abundant immune cell type in healthy lungs, play a key role in maintaining pulmonary homeostasis by phagocytizing and clearing foreign matter. After lung injury, PCECs recruit macrophages to perform the repair process by secreting diverse angiocrine factors. Two cohorts of patients with COVID‐19‐induced ARDS have shown that accumulation of CD163^+^ monocyte‐derived macrophages favors a profibrotic transcriptional phenotype.^84^ Some researchers support the notion that fibrosis is a macrophage‐driven process of excessive ECM accumulation. Macrophages stimulate pulmonary fibrosis by netrin1‐driven adrenergic processes.^85^ Itaconate is an endogenous antifibrotic factor in the lungs. In the bronchoalveolar lavage of IPF patients, itaconate level is reduced, and itaconate‐synthesizing cis‐aconitate decarboxylase (ACOD1) in airway macrophages decreases relative to controls.^86^ Bulk transcriptome, bulk DNA‐methylome, and single‐cell transcriptome results of peripheral blood samples harvested from COVID‐19 patients suggest an expansion of interferon‐activated circulating megakaryocytes (MKs) and increased erythropoiesis with characteristics of hypoxic signaling in severe COVID‐19.^87^ Leucine‐rich repeat kinase 2‐deficient AT2 cells in a classical bleomycin‐induced pulmonary injury model recruit profibrotic macrophages through the C‐C motif chemokine ligand2 (CCL2)/C‐C motif chemokine receptor 2 (CCR2) signaling pathway, causing more rapid and exacerbated fibrotic progression.^88^ Single‐cell RNA‐sequencing datasets of human liver and lungs reveal that CD9^+^ triggering receptor expressed on myeloid cells 2 (TREM2^+^) macrophages expressing secreted phosphoprotein 1 (SPP1), fatty acid binding protein 5 (FABP5), glycoprotein nmb (GPNMB), and CD63 are enriched at the outside edges of scarring and close to activated mesenchymal cells. These highly specific macrophages could serve as a clinical therapeutic target against fibrosis.^89^ The lungs from patients with different types of pulmonary fibrosis, including IPF, COVID‐19, and systemic sclerosis‐associated interstitial lung disease (ILD), and from mice with bleomycin‐induced pulmonary fibrosis are characterized by altered expression of methyl‐CpG‐binding domain 2 (MBD2) in macrophages. Following bleomycin induction in the lungs, MBD2 promotes TGF‐β1 production and M2 macrophage accumulation, causing severe fibrosis.^90^

In the early stage of lung injury, alveolar macrophages (AMs) migrate to the exposed position to capture and phagocytose inhaled pathogens and particles, without eliciting neutrophil influx and resulting in excessive inflammation.^9^, ^91^ When phagocytic function is exceeded, AMs trigger the inflammation process via the production of cytokines and chemokines that recruit and activate monocytes, neutrophils, and dendritic cells. In addition to the ability to promote inflammation, AMs are responsible for the secretion of TGF‐β, IL‐1RA, prostaglandins, and other immunomodulatory cytokines to solve inflammation.^92^, ^93^

Macrophages play an important and complex role in the repair and regeneration of lung injury. At different stages of disease progression, tissue‐resident macrophages and circulating monocytes/macrophages play different or even opposite regulatory roles through multiple mechanisms. Therefore, studying the regulatory role of macrophages in lung regeneration and fibrosis requires higher spatiotemporal resolution.

### 2.6 Neutrophils

Neutrophils are essential orchestrators of the human immune response against any type of pathogen or stimulus. The lungs are a major neutrophil reservoir. Abnormal activation of neutrophils leads to chronic lung inflammation.^94^, ^95^ The traditional view is that neutrophils exhibit terminal differentiation upon release from the bone marrow. With increased research, the functional diversity of neutrophils is now being recognized. Neutrophils acquire the ability to reprogram and adapt to the local microenvironment when recruited to damaged tissues.^96^

The occurrence of neutrophil precursors is the marker of severe COVID‐19.^97^ Neutrophils can be activated by epithelial cell necroptosis induced by nuclear mixed lineage kinase domain‐like pseudokinase (MLKL). Following a lethal dose of influenza A virus, mice lacking MLKL show reduced nuclear disruption of pulmonary epithelial cells, antagonized neutrophil recruitment into the infected lung, and increased survival rate.^98^ The release of neutrophil extracellular traps (NETs) is associated with the development of lung fibrosis, but the precise underlying mechanisms are not yet clear.^99^, ^100^, ^101^ In the mouse model of acute radiation injury to the lung, local activation of neutrophils is the key factor of the tumor‐supportive preconditioning of the lung microenvironment, modulated by the enhanced regenerative Notch signaling. Blocking degranulation by preventing neutrophil‐dependent Notch activation may significantly counteract radiation‐enhanced tumor cell metastasis.^102^ In addition to their direct involvement in the inflammatory response at the site of injury, neutrophils can regulate AM self‐renewal to maintain lung tissue homeostasis. Neonatal neutrophil‐derived 12‐hydroxyeicosatetraenoic acid (12‐HETE) is required for AM self‐renewal and maintenance during lung development. The deficiency of 12‐HETE results in a substantial decline in AMs in adult lungs and enhanced senescence. The compromised AM compartment is highly associated with increased susceptibility to LPS‐induced ALI and influenza A virus or SARS‐CoV‐2 lung infection.^103^ Single‐cell sequencing has revealed that pro‐neutrophils in the bone marrow have different transcriptional profiles from mature neutrophils migrating into inflamed mouse lungs. Neutrophils in the lungs of mice with pneumonia exhibit a predominance of Cxcr2, encoding receptors for the key neutrophil‐recruiting chemokines KC (CXCL1) and MIP2 (CXCL2).^104^ Another single‐cell cloning and single‐cell sequencing study has shown the presence of three variant progenitor cells in the distal airways of COPD patients, which may be responsible for neutrophilic inflammation, mucus hypersecretion, and fibrosis.^105^

As our understanding of the contribution of neutrophils to chronic disease improves and more data are obtained on the changes in the metabolic and transcriptional levels of neutrophils in disease states, neutrophil‐targeted therapies may soon become a new option for the treatment of diseases characterized by chronic neutrophilic inflammation, such as transfusion‐related ALI.

### 2.7 Platelets

Platelets are anucleate blood cells produced by MKs. Not only are they associated with hemostasis and thrombosis, but they are also involved in other physiological and pathological processes.^106^, ^107^ The bone marrow has been thought to be the site of MK maturation and platelet production, but recently, it has been shown that the lungs are the primary site of terminal platelet production, accounting for approximately 50% of total platelet production.^108^ Platelets are an important type of immune cell. Single‐cell sequencing results have shown that compared with bone marrow MKs, lung MKs express higher levels of immune molecules and have gene expression patterns similar to antigen‐presenting cells.^109^

According to public transcriptome datasets, the scores of MKs/platelets in the lungs and peripheral blood of patients with IPF present an opposite trend and are related to the disease progression. In mouse pneumonectomy (PNX) models, activated platelets stimulate SDF1 (known as CXCL12) receptors CXCR4 and CXCR7 on PCECs to guide the angiocrine membrane‐type metalloproteinase MMP14, potentiating alveolar epithelial cell expansion and neo‐alveolarization.^110^ The results suggest that hematopoiesis and lung platelet biogenesis serve as potential prognostic indicators in IPF.^111^ The animal models of lung fibrosis show that limiting inflammation is beneficial for disease progression. Unfortunately, the profit on animal encounter obstacles in clinical treatment.^10^ The core mechanism of pulmonary hypertension is inflammation. Platelets are involved in hypoxia‐driven inflammatory responses. In the mouse model of hypoxia‐induced inflammation, the number of platelets in the lungs is increased and activated. This process leads to an increase in the proinflammatory chemokines CXCL4 and CCL5, which in turn recruit more macrophages. This finding provides theoretical support for the role of platelets in inflammation‐mediated pulmonary hypertension and vascular remodeling.^112^ Deficiency or antagonism of protease‐activated receptor 4 reduces platelet aggregation, neutrophil recruitment, and NET production and protects mice from influenza A virus‐induced lung tissue damage and edema.^113^

In the state of homeostasis, the various cells in the lungs remain quiescent, maintaining a slow renewal. After lung damage, various cells are activated, proliferate, and differentiate into more mature cell lineages. In the process of lung repair, none of the cells are fighting alone but are regulated and coordinated by factors secreted by other surrounding cells. Therefore, when developing treatment methods, this issue should be considered, as treatment targeting a single target may have limited efficacy.

## 3 MOLECULAR MECHANISMS OF LUNG REGENERATION

The lungs are an organ with strong regenerative capacity in response to injury in mammals. There are many sources of lung stem/progenitor cells, which are usually in a silent state under steady‐state conditions.^114^ After lung injury, these cells could be elicited to proliferate and differentiate, supplement the damaged cell types, and maintain the normal physiological function of the lungs.^115^, ^116^ The development of single‐cell sequencing and lineage tracking provides an opportunity for researchers to deeply understand the origin and differentiation of cell subtypes.^117^, ^118^, ^119^, ^120^

### 3.1 Stem cell proliferation in lung regeneration

**FIGURE 2:**
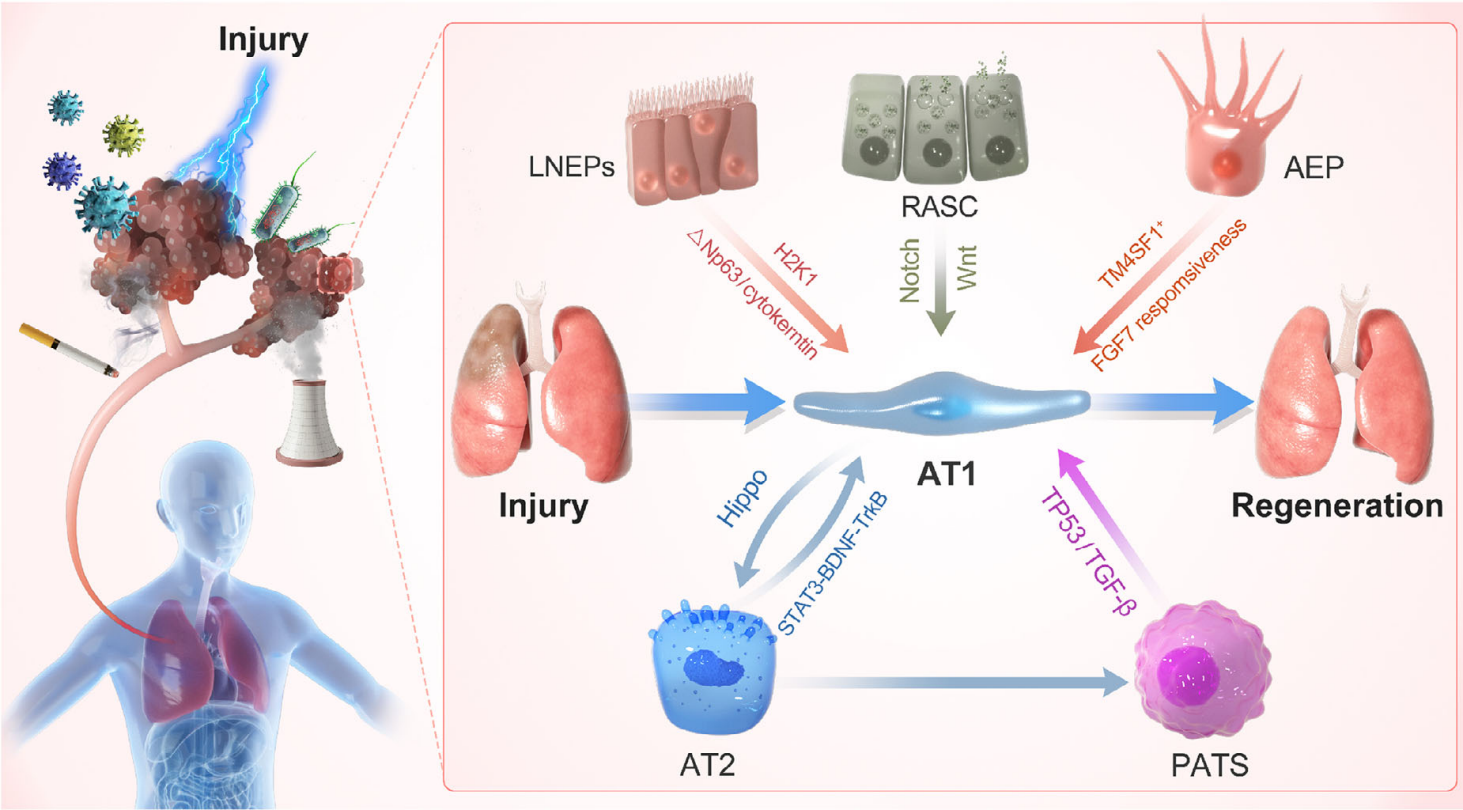
Sources of type I alveolar epithelial cells during lung regeneration. Type I alveolar epithelial cells are the specific units of lung tissue that perform the function of gas exchange. After injury, to rapidly restore lung function, in addition to type II alveolar epithelial precursor cells, other cell types of the respiratory system (such as bronchioalveolar stem cells and respiratory airway secretory cells) also rapidly transdifferentiate to supplement the number of AT1 cells. AEP, Wnt‐responsive alveolar epithelial progenitor; AT2, type 2 alveolar epithelial cell; H2‐K1, MHC class I marker; LNEPs, lineage negative epithelial progenitors; PATS, pre‐alveolar type‐1 transitional cell state; RASC, respiratory airway secretory cells; TM4SF1, transmembrane 4 L six family 1.

Genetic lineage tracing by dual recombinases has revealed that bronchioalveolar stem cells in response to different injuries can differentiate into multiple cell types, such as club cells, ciliated cells, AT1 cells, and AT2 cells.^121^ AT2 cells transdifferentiating into AT1 cells in primate lung tissue transiently pass through the intermediate state, which is different in mouse lungs.^24^ Stem/progenitor cell orthotopic transplantation is a potential treatment to promote lung regeneration. However, it is challenging to promote the functional proliferation of foreign stem cells in the receptor. Some studies have demonstrated that cotransplantation of lung progenitors and hematopoietic stem cells from the same donor can achieve durable tolerance without any chronic immune suppression.^122^ hepatocyte growth factor (HGF) derived from lung ECs has been testified to promote the proliferation of stem and parenchymal cells.^123^, ^124^ Three‐dimensional biomimetic porous collagen scaffold binding HGF maintains the biomimetic function of HGF to ameliorate the lung regeneration microenvironment. In a lung resection model in rats, implantation of a biomimetic scaffold with HGF can promote ECs and endogenous alveolar stem cells entering the scaffold at an early stage and attenuate inflammation and fibrosis at a later stage.^125^ Organoid models show that RAS cells in human respiratory bronchioles act as unidirectional progenitor cells for AT2 cells, which are fundamental for potentiating alveolar repair^32^ (Figure 2).

### 3.2 Tertiary lymphoid structure in lung regeneration

The immune system and tertiary lymphoid structure (TLS) are related to severe chronic inflammatory disease, particularly in the lung. Blockade of lymphotoxin β‐receptor signaling mitigates epithelial noncanonical activation of NF‐κB, quells TGF‐β signaling in the airway, and induces regeneration by protecting epithelial cells from death and activating WNT/β‐catenin signaling in alveolar epithelial stem/progenitor cells.^126^ Some patients who recover from COVID‐19 develop postacute sequelae of SARS‐CoV‐2, which affect their daily life to varying degrees. Mice adapted SARS‐CoV‐2 strain MA10 showed a TLS on lung histology examination 15−120 days after virus clearance, indicating that chronic inflammatory reactions still occur in the lungs after virus clearance.^127^ TLS has been found in lung tissue samples of patients with CF or non‐CF bronchiectasis. In mice, persistent airway infection with *P. aeruginosa* or *Staphylococcus aureus* triggers peribronchial TLS, but TLS germinal centers are absent in the lungs of lymphocyte‐depleted mice.^128^ Lymphatic follicles containing germinal centers are associated with various chronic airway diseases, such as COPD and severe asthma. In severe human COPD and COPD animal models, lymphatic follicles enhance the immune response to pulmonary pathogens. However, lymphatic follicular B cells may produce autoantibodies, which can perpetuate cigarette‐induced lung inflammation and damage.^129^ TLS is an organized infiltration of immune cells, which is closely related to the immunotherapy prognosis of lung tumors, such as non‐small‐cell lung cancer and lung adenocarcinoma.^130^, ^131^, ^132^ Epigenetic modulation also plays an important role in organ repair and regeneration.^34^, ^133^, ^134^ Pharmacological targeting of histone deacetylase 2 and DNA methyltransferase 1 can alleviate the degree of fibrosis.^34^ AT2 chromatin accessibility changes following injury, which is regulated by the STAT3–brain‐derived neurotrophic factor–tropomyosin receptor kinase B axis.^135^ During lung regeneration, the proliferation of epithelial cells is regulated by various cells in the surrounding environment. Lung regeneration is a complex process that involves coordinated regulation of multiple types of cells and molecules. Identifying key node molecules in this process and conducting targeted editing could help promote beneficial regeneration.

### 3.3 Single‐cell sequencing in lung regeneration

The lungs are an organ with a wide spatial diversity in architecture and cellular heterogeneity, making single‐cell sequencing technology a very important tool for studying lung repair and regeneration. Single‐cell sequencing technology has made it possible to know the gene expression patterns of individual cells in different pathophysiological states.^41^, ^136^ A growing number of open‐source tools have enabled us to predict the state, origin, and differentiation trajectory of cells, without the need for permanent labeling of the cells. By collecting samples at different time points for sequencing, we can accurately track the progression of the disease, which of course takes much time and money. Single‐cell sequencing technology allows us to gain a deeper understanding of cellular changes during lung repair, but there are still some obstacles. Spatial information is lost in the process of tissue digestion into single cells. The lung is a complex organ with different compartments and niches, and the same cell in different spatial locations has different roles, such as KRT5^+^ basal cells.^18^, ^62^, ^63^ Spatial transcriptome partially solves this problem, as spatial transcriptomes can be sequenced in situ on sections.^137^, ^138^, ^139^ However, the thickness and area of spatial transcriptome sections are limited.^140^, ^141^ Therefore, it is difficult to fully restore the information of the whole lung. It is hoped that this problem can be solved with the advancement of technology. Another approach is to sample different regions individually for sequencing. However, this operation increases the difficulty of subsequent data processing, and it is more difficult to define the edge parts of different regions in the diseased area. One of the hurdles to mapping the human lung using the single‐cell technique is the reproducibility of the results, possibly due to the different methods of tissue dissociation used by different subject groups. Therefore, to accomplish this goal, the same protocols need to be followed globally.

### 3.4 Uncontrolled repair process impair lung regeneration

After persistent or overwhelming damage, the repair process is out of control and tends to form fibrosis (known as scarring), which is characterized by excessive ECM deposition. Lung fibrosis is a shared feature of a variety of ILDs. It reduces the quality of life and leads to death from respiratory failure. A combination of aging, environmental, and genetic causes is involved in the activation of fibrotic processes, which probably begins many years before clinical manifestations. The knowledge of molecular and cellular mechanisms underlying fibrosis is fundamental for developing new clinical cures.

Aging‐reprogramed crosstalk between pulmonary capillary ECs, macrophages, and platelets leads to the loss of regenerative ability and favors fibrogenesis. Not only in the aging lungs but also in the liver and kidneys, ECs produce neuropilin‐1 (NRP1)/hypoxia‐inducible‐factor 2α (HIF2α) to suppress anti‐inflammatory and antithrombotic endothelial protein C receptor pathway. Reprogramed ECs secreting SDF1 recruit CXCR4^+^ TIMP1^high^ (tissue inhibitor of metalloproteinases 1) macrophages with platelets, which are activated to produce IL‐1α, to form the profibrotic platelet‐macrophage rosette. Blockade of NRP1 or HIF2α in ECs can restore the regenerative capacity of aging organs. Adoptive transfer of TIMP1^−/−^ monocytes or IL‐1α^−/−^ platelets can mitigate fibrosis of the damaged organs in old recipient mice.^142^

**FIGURE 3:**
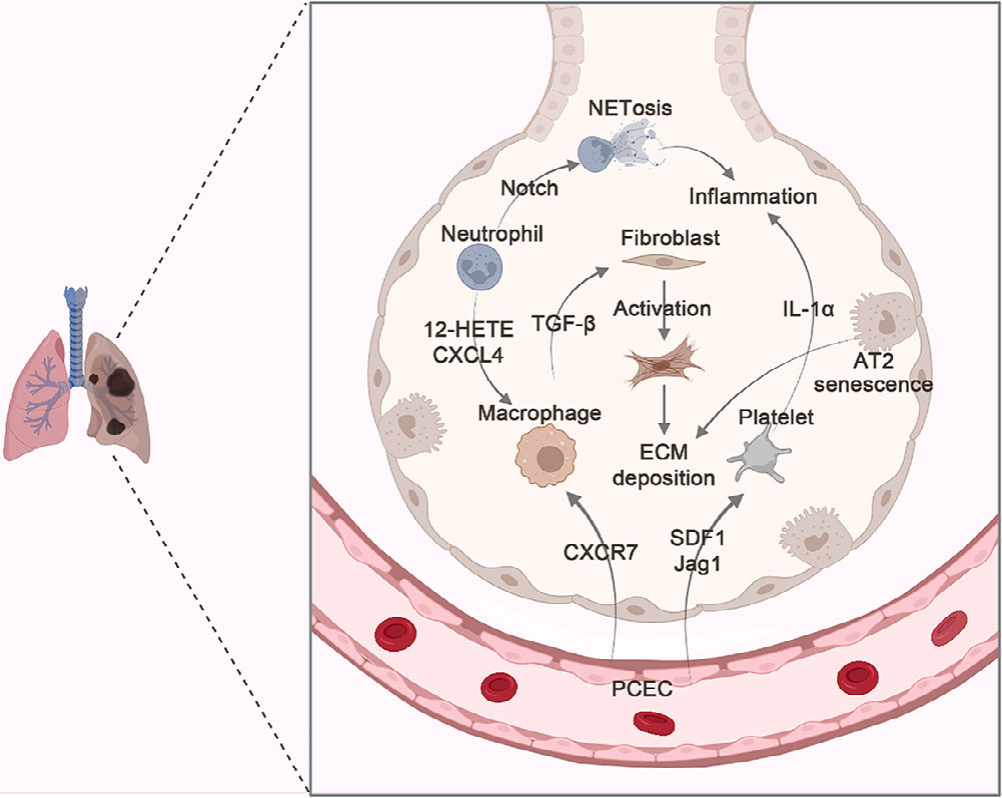
Cellular and molecular mechanisms in the process of pulmonary fibrosis. When the repair process loses control, multiple cell types, such as PCECs, macrophages, neutrophils, and platelets, are abnormally activated. The activated cells release various proinflammatory cytokines, activate fibroblasts, excessively secrete extracellular matrix, and destroy the structure and function of lung tissue. 12‐HETE, 12‐hydroxyeicosatetraenoic acid; AT2, type 2 alveolar epithelial cell; CXCR7, chemokine (C‐X‐C motif) receptor 7; CXCL4, platelet factor 4; ECM, extracellular matrix; Jag1, jagged canonical Notch ligand 1; SDF1, C‐X‐C motif chemokine ligand 12.

During the genesis of lung fibrosis, monocyte‐derived interstitial macrophages replace resident AMs to secrete profibrogenic factors. Mannosylated albumin nanoparticles incorporating TGF‐β small‐interfering RNA target the profibrotic subpopulation of CD206^+^ macrophages and prevent pulmonary fibrosis.^143^ EMT has been suggested as a relevant contributor to pulmonary fibrosis. In the lung tissue samples of IPF patients, low expression of miR‐200 promotes the high expression of beta‐tubulin‐III (Tubβ3), ZEB1, and β‐catenin and activates EMT, thereby aggravating fibrosis.^144^ In a mouse model, spermidine can alleviate bleomycin‐induced lung fibrosis by attenuating alveolar epithelial apoptosis and activating the endoplasmic reticulum stress (ERS)‐related pathway.^145^ Senescence rather than loss of AT2 cells promotes progressive lung fibrosis. Early blockade of senescence‐related signaling and elimination of senescent cells have high therapeutic value.^146^ methyl‐CpG bingding domain protein 2 (MBD2) deficiency in macrophages could attenuate TGF‐β1 production and reduce M2 macrophage accumulation in the lungs following bleomycin induction.^90^ IPF is a chronic progressive disease of unknown cause, in which fibrosis starts at the lung periphery and then progresses toward the lung center.^147^, ^148^, ^149^ Loss‐of‐function experiments have shown that *cell division cycle 42 (Cdc42)*‐knockout in AT2 cells causes periphery‐to‐center progressive pulmonary fibrosis.^150^ miR‐200c downregulates *Flt1* in ECs and induces the production of both soluble and ECM proteins, such as collagen type III alpha 1 chain (Col3a1), inter‐alpha‐trypsin inhibitor heavy chain 2 (Itih2), and serpin family C member 1 (SerpinC1), which in turn promote AT2 cell activation and transdifferentiation into AT1 cells^48^ (Figure 3). A deep understanding of the cellular and molecular mechanisms involved in the occurrence of pulmonary fibrosis can help to accurately develop clinical treatment strategies.

Transcriptomic studies across the adult range with accompanying structural and functional analyses of 86 human deceased donor lungs have shown that lung fibrosis is increasingly detected with aging and is related to poor outcomes in infection or ALI. p16 (CDKN2A), a canonical senescence marker, is the most highly upregulated gene in aging lungs. The average telomere length progressively decreases across the lifespan. Cellular composition analyses suggest that the proportion of epithelial cells decreases with age. However, the proportion of fibroblasts increases, accompanied by a fibrotic change in the aging lungs.^151^ Transcriptomic studies of human lung samples can identify novel genes and pathways involved in the human IPF, such as MMP, WNT pathway, epithelial genes, and microRNAs.^152^

## 4 RESEARCH MODELS OF LUNG INJURY AND REGENERATION

Animal models are an essential link for treatment from bench to bedside.^153^, ^154^, ^155^, ^156^ Pulmonary fibrosis has various or even multiple causes, especially IPF, the cause of which is unknown. However, genome‐wide association studies have shown the genetic variants that may contribute to the risk of IPF, such as telomerase reverse transcriptase (TERT), regulator of telomere elongation helicase 1 (RTEL1), KLF transcription factor 15 (KLF15), mitotic arrest deficient 1 like 1 (MAD1L1), SFTPC, and mucin 5B (MUC5B).^157^, ^158^, ^159^, ^160^ Animal models are one of the reasons that hinder the development of therapeutic methods for pulmonary fibrosis. Namely, different animal models have been used for research over the years. However, none of the animal models can completely simulate the clinical condition, especially the progressive and irreversible state. Each model can reflect part of clinical features.^161^ The human lungs in structure differ substantially from the murine lungs. In humans, the distal branches of the airway interweave with the alveolar gas exchange microenvironment, forming anatomical structures known as respiratory bronchioles, but this is not the case in mice.

### 4.1 Bleomycin‐induced lung injury model

Bleomycin‐induced animal models of pulmonary fibrosis are highly reproducible and highly restorative of the major histological features of the lungs of patients with pulmonary fibrosis. The bleomycin‐induced mouse lung injury model has multiple modes of administration, such as intraperitoneal (ip), intravenous (iv), intratracheal (it), and subcutaneous. Currently, the endotracheal route is the most common route of administration.^162^, ^163^, ^164^, ^165^ In mouse models, body weight‐based dosing typically ranges from 1.25 to 4 U/kg. Regarding the frequency of administration, bleomycin can be administered either as a single dose or as repeated low doses. Repeated dosing induces persistent fibrosis in mice, whereas fibrosis spontaneously regresses in mice that received a single dose.^166^, ^167^

Single administration of bleomycin directly to the lungs by endotracheal injection is widely used for studying lung fibrogenesis and evaluating antifibrotic therapy strategies. This animal model induces an early inflammatory response followed by interstitial fibrosis within 5−14 days.^168^, ^169^, ^170^ This model reflects a robust fibrotic response that displays several histological hallmarks seen in IPF patients.^166^ In mice, fibrosis can be reversed 3−4 weeks after intratracheal bleomycin injection.^164^, ^171^ Most of the data generated in preclinical studies for antifibrotic therapy include the use of the bleomycin‐induced animal model of pulmonary fibrosis. This research strategy for investigating IPF therapies has some limitations, particularly because the model used focuses on the fibrotic response following ALI rather than the progressive fibrosis observed in de novo IPF.^172^ For vascular ECs, there are different injury modes such as intravenous injection of oleic acid, LPS, and endotoxin.^173^ Some studies have shown a pioneering model of unilateral ALI, induced by cyclic rinsing with 0.9% saline and 0.3% Triton X‐100 in mechanically ventilated pigs.^174^

Genome‐wide association analysis has allowed researchers to recognize the contribution of genetic background to IPF, leading to the development of some genetically engineered mice to mimic the process of spontaneous pulmonary fibrosis. AT2 cell targeted knockout telomeric repeat binding factor 1 mice exhibit spontaneous pulmonary fibrosis characteristics through telomere damage.^175^, ^176^ Adenosine is a signaling nucleoside that is produced in response to cell injury and manages the balance between tissue protection and the progression of pathological tissue remodeling. Adenosine deaminase‐deficient mice show chronic lung disease features, such as progressive airway inflammation and airway remodeling, with elevated adenosine.^177^ In mouse models, abnormal expression of genes specifically expressed in the respiratory system, such as surfactant protein C (SFTPC) deficiency and mucin 5B (MUC5B) overexpression, can also cause spontaneous lung diseases similar to clinical phenotypes.^178^, ^179^, ^180^, ^181^

### 4.2 Left lung PNX model

Left lung PNX is a mature model for studying lung regeneration. Experimental PNX with good repeatability is significant to characterize the structural and physiological adaption of the lungs to the loss of functional gas‐exchange units. No inflammation occurs in the remaining lung tissue. Through this model, we can study the mechanism that drives the compensatory regeneration of the remaining lung tissue.^182^, ^183^

### 4.3 Human organoid model

Given many differences between animal and human lung physiology, the demand for human lung model systems has not been met. In vitro models can supplement animal models to improve our understanding of human lung physiology.^184^ Human organoid models are cost effective, personalized, and scalable models, which harbor both proximal and distal airway epithelium.^185^, ^186^, ^187^, ^188^ Organoids can be derived from various cell types of the lungs.^189^ The model infected with SARS‐CoV‐2 can recapitulate the transcriptomic signatures of patients’ samples.^190^ As previously mentioned, the lungs are a complex and dynamic organ, and the current 3D organ model cannot fully reflect the physiological process in the body. Although 3D lung organoid models have certain limitations, they are a promising useful tool for the study of human lung diseases.^191^, ^192^, ^193^, ^194^ A 3D organotypic culture system, where AT2 cells are grown in a complex collagen matrix in contact with fibroblasts, has been developed to study the mechanism of bronchopulmonary dysplasia.^195^

Another model widely used to study the physiology of the human respiratory system or drug delivery is the air–liquid interface method, which is advantageous to promote cell differentiation and sustain the histological and morphological features of airway epithelial cells.^196^ Organ‐type culture of primary human nasal epithelium differentiated at the air–liquid interface is a translation‐related primary cell model for the study of SARS‐CoV‐2 host–virus interaction, which has considerable potential in accelerating our understanding of pathogenesis.^197^, ^198^ Air–liquid interface culture systems can make up for the shortcomings of traditional cell culture systems, especially for pulmonary epithelial cells, which are closer to the real physiological state in vivo.^199^

Precision‐cut lung slice (PCLS) method is another commonly used method to study pathophysiologic processes in the lungs. Compared with cell culture, PCLS can realistically represent the 3D structure and cell populations of the lungs. Multiple sections can be prepared from a small piece of fresh tissue, thereby saving samples, but the culture time is limited to 7−10 days. This period can be extended to 21 days by changing the culture conditions. This method has some advantages over cell culture, but due to the limitation of the diffusion distance of nutrients in the culture medium, there is an upper limit to the thickness of the sections, which can only reflect part of the 3D structure of the lung.^200^, ^201^, ^202^

The differences in respiratory system structures and development between mice and humans make the collection and analysis of human clinical lung tissue samples important. In particular, the development of single‐cell transcriptome and spatial transcriptome also promotes the development of biological research from the hypothesis based on animal models of disease, with limited verification in human samples, to the hypothesis generation of unbiased molecular analysis based on human samples, and then verification using disease animal models. This transformation makes the biological research of lung diseases more efficient. Proteomic analysis of human lungs at 10 distinct time points from birth to 8 years indicates the existence of distinct molecular substages of alveolar development and predicts the age of independent human lung samples.^203^ Currently, the main clinical sample sources for studying lung diseases include biopsy samples, lung transplantation samples, autopsy samples, and alveolar lavage fluid.

Different methods of modeling can only represent certain features of respiratory diseases and do not fully replicate the complexity of human disease.^204^, ^205^ Yet, this is both a disadvantage and an advantage. Laboratory studies need to simplify complex problems in order to derive causal relationships.^206^ The bleomycin‐induced mouse model of pulmonary fibrosis is still the primary animal model for the study of lung disease, and the early molecular features induced by a single dose of bleomycin are the most similar to those of the acutely accelerated phase of human IPF. However, it should be noted that with a single injection, the disease phenotype in young mice spontaneously regresses, which differs somewhat from the clinical setting.^164^, ^207^ In addition to mice, there are large‐animal models for lung disease studies, such as dogs, pigs, and horses.^153^, ^208^ The respiratory system of large animals is anatomically closer to that of humans; however, there are disadvantages of a long period of modeling, difficulty in obtaining material, and a high mortality rate. We suggest that study models should be carefully selected and validated by at least two models to ensure the reliability of the study results.

## 5 THERAPEUTIC POTENTIAL OF LUNG REGENERATION IN LUNG DISEASE

Lung diseases such as COPD, IPF, and ARDS present high morbidity, mortality, and disability. Important advances in the last decade have allowed a better understanding and characterization of these diseases, and some therapeutic approaches have been proposed. Nevertheless, there is still a long way to go in terms of accurate diagnosis and complete cure. Researchers and physicians are also actively exploring new treatments, such as stem cell transplantation.

### 5.1 Chronic obstructive pulmonary disease

#### 5.1.1 Overview of COPD

COPD—a chronic respiratory disease with sputum, cough, asthma, inflammation, and dyspnea as its main symptoms—is a common disorder in the elderly, and cigarette smoking is the main risk factor for COPD development in genetically susceptible individuals.^209^, ^210^ COPD is also a trigger for other organ diseases. This disease is easily ignored because patients are elderly people and the symptoms of dyspnea are often attributed to functional decline or old age. Pathological staining shows alveolar epithelial cell death, disorganization of ECM in the alveolar region, and alveolar collapse. Anatomical results show mucus retention and airway wall remodeling in the respiratory tract.^211^ Many elderly patients with severe asthma, especially those with a history of smoking, show typical clinical features of COPD, including irreversible airway obstruction, known as “asthma–COPD overlap syndrome.” COPD exacerbations are featured by cough, shortness of breath, and mucus production beyond baseline day‐to‐day variation. These detrimental events lead to accelerated lung function decline, increased cardiac events, and mortality.^212^ Prevention of acute exacerbations is essential in the management of COPD.^213^

#### 5.1.2 Clinical and potential therapeutic interventions in COPD

The current goal of drug therapy for COPD patients is to alleviate symptoms, improve exercise tolerance, and reduce the risk of worsening the condition. Currently, the treatment of symptomatic COPD patients is usually targeted based on the progression of the disease. The initial treatment mainly includes bronchodilators, such as long‐acting muscarinic antagonists.^214^, ^215^ The results of a 52‐week randomized clinical trial have shown that triple therapy with twice‐daily inhaled glucocorticoid (160 or 320 μg of budesonide), long‐acting muscarinic antagonist (18 μg of glycopyrrolate), and long‐acting β2‐agonist (9.6 μg of formoterol) causes a lower rate of moderate‐to‐very‐severe COPD exacerbations than glycopyrrolate–formoterol or budesonide–formoterol.^216^ Progressive destruction of alveolar tissue is one of the hallmarks of COPD, so there is increasing research on regenerative therapy based on stem cells and their derived products for repairing alveolar structure and restoring function.^217^ A study of a rat COPD model induced by cigarette exposure has shown that human umbilical cord mesenchymal stem cell (hUC‐MSC)‐derived extracellular vesicles (EVs) are effective in ameliorating COPD‐induced inflammation. Compared with the control group, both the transplantation of hUC‐MSCs and the application of EVs ameliorate peribranchial and perivascular inflammation, decrease alveolar septal thickening associated with mononuclear inflammation, and reduce the number of goblet cells.^218^

In addition to animal experiments, clinical studies of stem cell transplantation are being conducted in various countries (Table 1). The 12‐month follow‐up of late‐stage COPD patients receiving bone marrow‐derived monocytes (BM‐MCs) through intravenous injection has shown no adverse reactions; meanwhile, within 30 days after surgery, the patients’ lung function has slightly improved. The purpose of that study was to evaluate the safety of BM‐MC transplantation, and the results were gratifying. That study had a small sample size (only four patients) and lacked clear results with statistical analysis. However, the study has laid the foundation for other cell transplantation experiments in the future.^219^, ^220^ A subsequent clinical trial of 62 patients undergoing in vitro culture screening of bone marrow‐derived mesenchymal stem cells has shown that intravenous infusion of allogeneic mesenchymal stem cells is safe during a 2‐year follow‐up period. The significant decrease in C‐reactive protein levels indicates that MSCs can to some extent reduce systemic inflammatory response. Unfortunately, no statistically significant differences were observed in lung function and quality of life. However, sufficient cases provide more confidence in the safety of MSC therapy for COPD.^221^ Intravenous infusion of stem cell concentration needs to be precisely controlled, with a too low cell volume causing a poor therapeutic effect and a too high cell volume easily causing pulmonary embolism. The residence time of intravenous infusion in the lungs is not long enough for effective action. Intratracheal administration has become a new mode of drug delivery. Due to the large surface area of the alveoli, high membrane permeability, and low enzyme activity in the lungs, this delivery method theoretically has a higher targeting and success rate.^222^, ^223^

**TABLE 1:** Part of clinical trials of stem cell treatment for COPD patients from 2010 to 2024.

Clinical trial identifier	Intervention	Clinical phase	Study completion	Enrollment	Sponsor
NCT00683722	Prochymal™	Phase 2	2010‐08	62	Mesoblast, Inc.
NCT01110252	Stem cells stimulation	Not Applicable	2011‐11	4	UPECLIN HC FM Botucatu Unesp
NCT01110252	Stem cells stimulation	Not Applicable	2011‐11	4	UPECLIN HC FM Botucatu Unesp
NCT02645305	Adipose derived stem cells	Phase 1/ Phase 2	2016‐12	20	University of Science Ho Chi Minh City
NCT02412332	Bone marrow‐derived stem cells	Phase 1/ Phase 2	2017‐04	20	UPECLIN HC FM Botucatu Unesp
NCT02216630	Adipose derived stem cells	Phase 1/ Phase 2	2017‐07	26	Kimera Society Inc
NCT03092648	Bronchial basal cells	Phase 1/ Phase 2	2019‐02	20	Nanfang Hospital, Southern Medical University
NCT03188627	Bronchial basal cells	Phase 1/ Phase 2	2019‐12	24	Regend Therapeutics
NCT03655795	Bronchial basal cells	Phase 1	2021‐03	20	Ruijin Hospital
NCT03021681	Bronchial basal cells	Not Applicable	2021‐11	18	Huai'an No.1 People's Hospital
NCT05594303	Bronchial basal cells	Phase 1/ Phase 2	2022‐11	6	Guangzhou Institute of Respiratory Disease
NCT04433104	Umbilical cord mesenchymal stem cells	Phase 1/ Phase 2	2021‐12	40	Vinmec Research Institute of Stem Cell and Gene Technology
NCT03156673	Bronchial basal cells	Early Phase 1	2022‐06	20	First Affiliated Hospital of Shantou University Medical College
NCT04047810	Mesenchymal stem cells	Phase 1	2023‐12	15	Mayo Clinic
NCT05638776	REGEND001	Phase 2	2024‐06	50	Regend Therapeutics
NCT04206007	UMC119‐06	Phase 1	2024‐06	9	Meridigen Biotech Co., Ltd.

REGEND001, a cell therapy product made from bronchial basal cells with the ability to regenerate lung tissue; UMC119‐06, ex vivo cultured human umbilical cord tissue‐derived mesenchymal stem cells product.

*Data sources*: clinical trials website, https://clinicaltrials.gov/.

COPD is currently an incurable disease that reduces the quality of life of patients to varying degrees. However, through the active exploration of researchers and clinical doctors, there are currently many treatment methods undergoing clinical trials. Their safety and effectiveness have also achieved some preliminary encouraging results. However, there is still a long way to go in the future.

### 5.2 Idiopathic pulmonary fibrosis

#### 5.2.1 Overview of IPF

IPF, a progressive age‐related disease, is a fatal fibrosing ILD with a mean survival time of 3−5 years after diagnosis.^224^, ^225^, ^226^ The histopathological results of IPF show subepithelial fibrosis foci, subpleural fibrosis, and microscopic honeycombing. The abnormal regions are usually connected with the histopathologically normal areas.^227^ IPF pathogenesis involves chronic injury to alveolar epithelial cells and the dysregulation of subsequent repair procedures, resulting in scarring in the lungs and hampering pulmonary function.^228^ While the correlation between aging and dysregulated wound repair in IPF is beyond doubt, the underlying molecular and cellular mechanisms still require more evidence.^229^

#### 5.2.2 Clinical and potential therapeutic interventions in IPF

To date, lung fibrosis has limited therapeutic options, mainly including chemical drugs and organ transplantation. Nintedanib and pirfenidone are the only two drugs approved by the US FDA for the treatment of IPF. These two drugs can reduce the rate of decline in lung function and slow fibrosis progression; however, they cannot stop or reverse disease progression. In addition, both compounds may have varying degrees of side effects, such as nausea and diarrhea. Lung transplantation, which is limited by the donors and high costs, cannot be an effective treatment for lung disease. Therefore, it is urgent to develop new therapies for pulmonary fibrosis.^230^ The current research focuses on developing new therapies that not only target abnormal deposition of ECM but also target other signaling pathways such as vascular endothelial angiocrine factors and host immune response‐mediated signaling pathways.^231^, ^232^ Several clinical trials of biological agents such as recombinant proteins, growth factor antibodies, prostacyclin analogs, protein precursors, and cell‐based or cellular products‐based therapies for the treatment of IPF are ongoing. PRM‐151 is a recombinant human pentraxin 2 protein that inhibits the migration of profibrotic monocyte‐derived AMs into fibrotic area.^233^ The phase 2 and phase 3 clinical trials for PRM‐151 have been completed, mainly to evaluate its safety and effectiveness in treating mild to moderate IPF patients. Phase 2 results showed that IPF patients receiving recombinant protein showed a slower rate of decline in lung function than the comfort group, with the main adverse events being cough, fatigue, and nasopharyngeal inflammation.^225^ Unfortunately, the phase 3 clinical trial was prematurely stopped due to futility after having enrolled 655 IPF patients with less functional benefit than severe adverse effects.^234^ Connective tissue growth factor (CTGF) is a mediator of tissue remodeling, which acts on connective tissue cells downstream of TGF‐β and stimulates fibroblast proliferation and ECM production. Pamrevlumab is an anti‐CTGF antibody that is studied for the treatment of IPF. Unfortunately, the phase 3 study on pamrevlumab was not effective at preserving lung function in patients with IPF.^234^

Cell therapy with stem cells is a promising therapeutic alternative with great potential for applicability. The regenerative approach for the lung aims to repair injured tissue through the delivery of either stem cells or products of stem cells that boost lung regeneration. Due to their broad range of sources and ability to differentiate and regenerate, MSCs are widely used in clinical practice. Clinical trials have revealed that a high cumulative dose of MSCs delivered through intravenous infusion is safe and well tolerated by IPF patients with a rapid lung function decline (Table 2). During the intervention period, the patients showed increased lung function.^235^

**TABLE 2:** Part of clinical trials of stem cell treatment for IPF patients from 2013 to 2025.

Clinical trial identifier	Intervention	Clinical phase	Study completion	Enrollment	Sponsor
NCT01385644	Placental mesenchymal stem cells	Phase 1	2013‐05	8	The Prince Charles Hospital
NCT02135380	Autologous stromal vascular fraction	Phase 1/ Phase 2	2015‐08	60	Kasiak Research Pvt. Ltd.
NCT02013700	Allogeneic adult human mesenchymal stem cells	Phase 1	2016‐11	9	Joshua M Hare
NCT01919827	Adult mesenchymal stem cells	Phase 1	2018‐05	17	Clinica Universidad de Navarra, Universidad de Navarra
NCT02277145	Umbilical cord mesenchymal stem cells	Phase 1	2018‐12	10	Jianwu Dai
NCT02745184	Lung stem cells	Phase 1/ Phase 2	2020‐08	10	Shanghai East Hospital
NCT04262167	Lung spheroid stem cells	Phase 1	2024‐10	24	University of North Carolina, Chapel Hill
NCT06081621	REGEND001	Phase 2	2024‐12	20	Regend Therapeutics
NCT05468502	Human umbilical cord mesenchymal stem cell	Phase 1	2025‐08	18	Shanghai Life Science & Technology
NCT05016817	AlloRx	Phase 1	2025‐09	20	The Foundation for Orthopaedics and Regenerative Medicine

AlloRx, cultured allogeneic adult umbilical cord‐derived mesenchymal stem cells; REGEND001, a cell therapy product made from bronchial basal cells with the ability to regenerate lung tissue.

*Data sources*: clinical trials website, https://clinicaltrials.gov/.

### 5.3 Acute respiratory distress syndrome

#### 5.3.1 Overview of ARDS

ARDS is a common cause of respiratory failure in critically ill patients with a high mortality rate of 30−40%.^236^ Pathological results from patients with ARDS often show diffuse alveolar injury. Laboratory studies have shown that alveolar epithelium and pulmonary endothelium are damaged, leading to the accumulation of protein‐rich inflammatory edema fluid in the alveolar cavity.^237^ SARS‐CoV‐2 is a highly pathogenic and transmissible virus that mainly attacks the respiratory system and causes varying degrees of lung function damage. Several clinical cohort studies indicate that age is the leading risk factor for severe outcomes in patients with COVID‐19.^238^ The pathological results show hyaline membrane formation, bilateral diffuse alveolar damage, apoptosis and desquamation of pneumocytes, and deposition of ECM in the lungs of patients with COVID‐19.^238^ Dyspnea is the most common persistent symptom among COVID‐19 survivors. Focal fibroproliferative diffuse alveolar damage is seen in samples from explanted lungs and autopsy specimens. Myofibroblast proliferation, microcystic honeycombing, and mural fibrosis also occur in rare areas. Even 20−30% of patients with COVID‐19 show vascular microthrombosis and macrothrombosis in the lungs. Meanwhile, widespread thrombosis with microangiopathy and endothelial injury in the lungs is seen at autopsy.^239^, ^240^, ^241^, ^242^ ARDS also brings a significant long‐term disability burden, with only 50% of survivors being able to return to work at 1 year.^243^

#### 5.3.2 Clinical and potential therapeutic interventions in ARDS

Despite years of research, there are currently no disease‐modifying therapies for ARDS. The use of lung‐protective ventilation, prone positioning, and muscle relaxants has improved survival in patients with moderate to severe ARDS over the past decade. After the shock subsides, liquid conservative treatment can increase the number of days without ventilation.^244^, ^245^ Countless drugs that have shown promise in preclinical studies have been ineffective in human trials, a gap attributed in part to the heterogeneity of clinical risk factors, lung injury physiology, microbiology, and biology of human ARDS.^246^ Therefore, a more cautious approach is needed for the treatment of SARS‐CoV‐2‐related ARDS.

Drug repurposing is one of the potential therapeutic options for IPF that have long been considered. It is a strategy aimed at identifying new indications and targets for approved or studied drugs that exceed their original medical indications, which can save development costs and reduce development times.^247^ Due to the certain connections between different diseases, similar pathogenesis, targets, or causes, researchers classified and screened marketed drugs based on these characteristics or commonalities, and conducted targeted preclinical studies on many marketed drugs, providing a theoretical basis for the treatment of pulmonary fibrosis. A successful example of drug repurposing is the US FDA's approval of remdesivir (Veklury®; Gilead Sciences) for the treatment of COVID‐19.^248^ In addition, many US FDA‐approved drugs for other diseases are being used to treat lung diseases in clinical trials, including but not limited to antitumor drugs imatinib and anlotinib; cardiovascular drugs losartan and macitentan; antibiotics doxycycline and minocycline; metabolic disease drug metformin; and neurological drug melatonin. Safety and effectiveness are issues that researchers must consider when designing “repurposed drugs”; therefore, sufficient preclinical and clinical trials are necessary. Regulatory and patent considerations and the limited efficacy of repurposed drugs are factors in successful drug repurposing.

MSCs‐derived exosomes (MSC‐Es) can reverse LPS‐induced ALI through downregulation of nuclear factor erythroid 2‐related factor 2 and antioxidant‐response element factors.^249^ Although the mechanism underlying the regulation of MSC‐Es remains unclear, the outcomes are generally thought to be related to the miRNA cargo and tissue‐specific proteins. We summarize some clinical trials that used stem cells or their products for the treatment of ARDS in Table 3. Due to the ability to package various therapeutic agents with varying size range and low immunogenicity, MSC‐Es have been considered a potential candidate for drug delivery and combination therapy.

**TABLE 3:** Part of clinical trials of stem cell treatment for ARDS patients from 2014 to 2026.

Clinical trial identifier	Intervention	Clinical phase	Study completion	Enrollment	Sponsor
NCT01902082	Mesenchymal stem cells	Phase 1	2014‐06	20	Shaoxing Second Hospital
NCT02215811	Mesenchymal stromal cells	Phase 1	2015‐12	10	Karolinska University Hospital
NCT0209764	Allogeneic bone marrow‐derived human mesenchymal stromal cells	Phase 2	2018‐02	60	Michael A. Matthay
NCT02804945	Mesenchymal stem cells	Phase 1	2019‐06	20	M.D. Anderson Cancer Center
NCT04525378	Mesenchymal stromal cell‐based therapy	Phase 1	2020‐10	20	D'Or Institute for Research and Education
NCT03608592	Umbilical cord derived mesenchymal stem cells	Not Applicable	2020‐12	26	Sun Yat‐sen University
NCT04366063	Mesenchymal Stem Cells	Phase 2/ Phase 3	2020‐12	60	Royan Institute
NCT04400032	Mesenchymal stromal cells	Phase 1/ Phase 2	2021‐04	15	Ottawa Hospital Research Institute
NCT04493242	ExoFlo	Phase 2	2021‐05	102	Direct Biologics, LLC
NCT04625738	Mesenchymal stem cells	Phase 2	2021‐09	30	Central Hospital, Nancy, France
NCT04333368	Umbilical cord derived mesenchymal stem cells	Phase 1/ Phase 2	2021‐10	47	Assistance Publique—Hôpitaux de Paris
NCT04399889	Human cord tissue mesenchymal stromal cells	Phase 1/ Phase 2	2022‐02	12	Joanne Kurtzberg, MD
NCT04865107	Umbilical cord‐derived mesenchymal stromal cells	Phase 2	2022‐04	54	Ottawa Hospital Research Institute
NCT04390152	Mesenchymal stem cells	Phase 1/ Phase 2	2022‐04	40	BioXcellerator
NCT04602104	hMSC‐Exos	Phase 1/ Phase 2	2022‐09	169	Ruijin Hospital
NCT04456361	Mesenchymal stem cells	Early Phase 1	2022‐12	9	Instituto de Medicina Regenerativa
NCT04390139	XCEL‐UMC‐BETA	Phase 1/ Phase 2	2022‐12	26	Banc de Sang i Teixits
NCT04345601	Mesenchymal stromal cells	Phase 1/ Phase 2	2023‐01	28	Baylor College of Medicine
NCT04377334	Mesenchymal stem cells	Phase 2	2023‐02	40	University Hospital Tuebingen
NCT04494386	Umbilical cord lining stem cells	Phase 1/ Phase 2	2023‐07	17	Restem, LLC.
NCT05387278	EV‐Pure™ and WJ‐Pure™	Phase 1	2023‐12	20	Vitti Labs, LLC
NCT04466098	Mesenchymal stromal cells	Phase 2	2023‐12	9	Masonic Cancer Center, University of Minnesota
NCT04565665	Mesenchymal stem cell	Phase 1/ Phase 2	2024‐04	70	M.D. Anderson Cancer Center
NCT04452097	Human umbilical cord mesenchymal stem cells	Phase 1/ Phase 2	2024‐05	39	Baylx Inc.
NCT03818854	Human mesenchymal stromal cells	Phase 2	2024‐07	120	Michael A. Matthay
NCT05354141	ExoFlo	Phase 3	2024‐08	970	Direct Biologics, LLC
NCT05127122	Bone marrow mesenchymal stem cell derived extracellular vesicles	Phase 1/ Phase 2	2024‐12	81	Direct Biologics, LLC
NCT04447833	Mesenchymal stromal stem cells	Phase 1	2025‐06	7	Uppsala University
NCT04347967	UMC119‐06	Phase 1	2025‐09	18	Meridigen Biotech Co., Ltd.
NCT05983627	Human umbilical cord mesenchymal stem cells	Phase 1	2026‐12	9	Asia Cell Therapeutics (Shanghai) Co., Ltd.

Abbreviations: EV‐Pure™, placental‐derived exosomes; ExoFlo, bone marrow mesenchymal stem cell‐derived extracellular vesicles; ExoFlo, bone marrow‐derived extracellular vesicles; hMSC‐Exos, allogeneic human mesenchymal stem cell exosomes; UMC119‐06, human umbilical cord‐derived mesenchymal stem cells; WJ‐Pure™, umbilical cord mesenchymal stem cells; XCEL‐UMC‐BETA, Wharton Jelly mesenchymal stromal cells.

Data sources: clinical trials website, https://clinicaltrials.gov/.

Safety is the first consideration for different treatment methods, followed by clinical outcomes. It is necessary to improve effectiveness while reducing side effects.^250^, ^251^ The issues that need to be considered in stem cell transplantation are the administration method, time, and dosage.^252^ Research has shown that multiple doses have better therapeutic effects than single doses.^253^, ^254^ In addition to intravenous infusion and tracheal delivery, inhalation is a milder and painless method. However, it is necessary to consider the decrease in cell activity of stem cells as they pass through the respiratory tract. Additionally, attention should be paid to the dosage. High‐dose stem cells undergo uncontrolled proliferation and differentiation in the body, which carries the risk of developing cancer.^255^ Another issue that needs to be noted is the directional differentiation of stem cells after transplantation to the damaged site. It is a challenge to induce stem cells to differentiate into functional lung epithelial cells. The combination of stem cell transplantation and small‐molecule preparations may be an effective method to solve this problem. After stem cell transplantation, small‐molecule preparations are delivered to the damaged site to induce targeted differentiation of stem cells.

## 6 CONCLUSIONS

In the past decade, researchers and government institutions around the world have invested considerable attention and financial resources into the field of lung regeneration and fibrosis. Several therapeutic targets have been identified, and a large portfolio of new compounds has been developed, which are currently undergoing clinical trials. However, there is still no effective treatment that can completely cure or reverse pulmonary fibrosis. The development of cell‐specific lineage tracing and single‐cell sequencing with more animal models has allowed researchers to precisely know the definition of all sub‐cell types, providing more accurate targets for clinical therapy. Specially, the ongoing COVID‐19 pandemic has necessitated research on lung injury repair. An in‐depth study of the molecular and cellular mechanisms underlying lung fibrosis is essential.

Stem/progenitor cell transplantation is currently a promising treatment for lung diseases. However, significant translational challenges remain. Poor efficiency of in vivo stem cell expansion in recipient lesions is the major roadblock. Promoting the functional proliferation of endogenous or foreign stem cells in the recipient organ is fundamental. Accumulating evidence demonstrates that angiocrine factors derived from ECs might be crucial for the safe physiological self‐renewal of engraftable stem cells, a procedure that cannot be replicated by the present methods. It is rational to believe that the regeneration potential and homeostasis of almost every organ in mammals are modulated by angiocrine factors. Whether based on stem cells or chemical drugs to treat lung diseases, it is necessary to deepen our understanding of the mechanisms within various types of cells in the lung and the communication between them. The rapid development of biotechnology means that we are at the onset of realizing the promise of lung regenerative medicine.

Currently, there is a probability of delayed diagnosis and misdiagnosis in the diagnosis of lung diseases. This is partly due to the similar symptoms of different lung diseases, such as difficulty breathing, coughing, and inflammation. These diagnostic difficulties have brought some difficulties to subsequent treatment. Therefore, cross‐validation of multiple diagnostic methods such as hematology, pathology, and imaging is required. More importantly, it is necessary to develop more sensitive and convenient molecular markers and clinical diagnostic methods. The idea of precision medicine needs to be carried through from diagnosis to the end of treatment.

The field of lung repair and regeneration has made significant progress in the past decade. The development of single‐cell sequencing, spatial transcriptomics, and lineage tracking technology provides an opportunity for researchers to deeply explore molecular mechanisms. A variety of damage models and higher‐resolution imaging technology can enable us to better simulate different clinical‐pathological states. Organoid and PCLSs allow for the direct use of human tissue for experimentation and evaluation, compensating for the evolutionary differences between humans and experimental animals. Based on urgent practical needs, some innovative clinical trials are being conducted on a large scale, and there is still great room for improvement in treatment effectiveness.

## AUTHOR CONTRIBUTIONS

Y. C., C. M., and B. D. conceived and designed the work. Y. C., C. M., and B. D. wrote and revised the manuscript. Z. L., G. J., and S. W. revised the manuscript. All authors contributed to the article, read, and approved the final manuscript.

## CONFLICT OF INTEREST STATEMENT

All authors have no conflict of interest to declare.

## ETHICS STATEMENT

Ethics statement was waived because it was a review.

## Data Availability

All data generated or analyzed during this work are included in this published review.
